# TRENDS IN MEDICARE BENEFICIARY PAYMENTS FOR NONHOSPICE SERVICES DURING HOSPICE ELECTIONS

**DOI:** 10.1093/geroni/igae098.0751

**Published:** 2024-12-31

**Authors:** Thomas Christian, Michael Plotzke

**Affiliations:** Abt Associates, Cambridge, Massachusetts, United States; Abt Associates, St. Louis, Missouri, United States

## Abstract

Hospice election marks a shift towards palliative objectives, although beneficiaries retain curative coverage for unrelated (non-terminal) conditions. Little is known about beneficiary cost sharing for these services. Using Federal Fiscal Year (FY) 2022 Medicare claims, we cross-referenced beneficiaries’ hospice and non-hospice services claims’ dates. For overlapping claims, we totaled beneficiaries’ liability payments (coinsurance and deductibles) by year, claim type, and ICD-10 code. We used linear regression analysis to assess trends with beneficiary and hospice characteristics. We also compared beneficiary payments to Consumer Assessment of Healthcare Providers & Systems (CAHPS) Hospice score star ratings. In FY2022, Medicare paid $931.1 million for non-hospice services during hospice election, for which beneficiaries were collectively billed $206.8 million (18.2%), with 45.0% of hospice beneficiaries paying some amount. The majority was for services on or after the 60th day from hospice admission. Part B accounted for most (88.9%) of the total beneficiary payments. Pressure ulcers, end stage renal disease, hypertension, and COVID-19 were associated with the greatest payment amounts. Beneficiary payments were largest among beneficiaries black or Hispanic, or younger, or terminal heart disease, or dual-eligible for Medicaid, or in a nursing facility, or from a newer, for-profit, or southern hospice. Daily payment rates were $0.32 at the 10th percentile and $3.31 at the 90th percentile. Daily payment rates were $2.17 at CAHPS one-star hospices vs. $1.00 at five-star hospices. CMS should monitor these trends to ensure that vulnerable beneficiaries are not unfairly impacted and evaluate whether beneficiary payments are a useful hospice quality indicator.

